# Recognition of child maltreatment in emergency departments in Europe: Should we do better?

**DOI:** 10.1371/journal.pone.0246361

**Published:** 2021-02-05

**Authors:** F. Hoedeman, P. J. Puiman, A. W. Smits, M. I. Dekker, H. Diderich-Lolkes de Beer, S. Laribi, D. Lauwaert, R. Oostenbrink, N. Parri, L. García-Castrillo Riesgo, H. A. Moll

**Affiliations:** 1 Department of General Paediatrics, Erasmus MC-Sophia Children’s Hospital, Rotterdam, The Netherlands; 2 Augeo Foundation, Driebergen, The Netherlands; 3 Emergency Department, Medical Center Haaglanden, The Hague, The Netherlands; 4 Emergency Department, Tours University Hospital, Tours, France; 5 Emergency Department, University Hospital Brussels (UZ Brussel), Brussels, Belgium; 6 Emergency Department & Trauma Center, Ospedale Pediatrico Meyer Firenze, Florence, Italy; 7 Emergency Department, University Hospital Marques de Valdecilla, Cantabria, Spain; Federal University of Sergipe, BRAZIL

## Abstract

**Objectives:**

To evaluate the different policies to recognize child maltreatment in emergency departments (EDs) in Europe in order to define areas of improvement.

**Methods:**

A survey was conducted on the recognition of child maltreatment in EDs in European countries with a focus on screening methods, parental risk factors, training and hospital policies. The survey was distributed through different key members from the EUSEM, REPEM and the EuSEN. A summary score based on the NICE guideline (4 questions on child characteristics, 4 questions on parental characteristics and 5 questions on hospital policy) was calculated.

**Results:**

We analysed 185 completed surveys, representing 148 hospitals from 29 European countries. Of the respondents, 28.6% used a screening tool, and 31.8% had guidelines on parental risk factors. A total of 42.2% did not follow training based on child characteristics, and 57.6% did not follow training on parental characteristics. A total of 71.9% indicated that there was a need for training. 50.8% of the respondents reported a standardized policy for the detection of child maltreatment. Translating the survey results to NICE summary scores of the EDs in Europe, we found that 25.6% (34/133) met most, 22.6% (30/133) met some and 51.9% (69/133) met few of the NICE guideline recommendations. More specifically, with respect to hospital policies, 33.8% (45/133) met most, 15.0% (20/133) met some and 51.1% (68/133) met few of the NICE guideline recommendations.

**Conclusion:**

There is high variability regarding policies for child maltreatment detection and only a quarter of the EDs met most of the NICE guideline recommendations for child maltreatment. There is a need for the use of screening tools, training of ED staff and implementation of local hospital policies.

## Introduction

Child maltreatment is a major public health problem and responsible for a huge socio-economic burden [1,2]. The World Health Organization (WHO) defines child maltreatment as the abuse and neglect that occurs to children under 18 years of age; it includes all types of physical and/or emotional ill-treatment, sexual abuse, neglect, negligence and commercial or other exploitation, which results in actual or potential harm to the child’s health, survival, development or dignity in the context of a relationship of responsibility, trust or power [3]. In 1989, the United Nations developed the Convention on the Rights of the Child, which states that all appropriate actions shall be taken to protect children from any form of physical, mental or sexual abuse and/or neglect [4,5]. However, in Europe child maltreatment today is estimated to affect 117 million children under 18 years of age and to cause approximately 850 deaths per year of children younger than 15 years [2]. It was suggested that difficulties in recognizing child maltreatment by paediatricians, among other reasons, even lead to underestimation of the numbers of victims and fatal cases [2]. The negative impact of child maltreatment on individual health is well known and the long lasting effects persist into adulthood varying from mental health problems, like substance abuse, depression, psychological distress and suicide to physical health problems, like respiratory disease, chronic pain, obesity, memory impairment and even ischemic heart disease [6–10]. The stress response provoked by situations of maltreatment in childhood results in impairment in multiple structures and functions of the brain [8,11]. A recent study suggests that child maltreatment may even impact the offspring of child maltreatment victims by epigenetic changes in spermatozoal DNA [12]. Reducing the occurrence of child maltreatment and early intervention after child maltreatment may prevent fatal outcomes and improve the health related quality of life in adulthood [6,13]. Unfortunately, child maltreatment remains difficult to identify, because it is often not the primary reason for a child to visit a doctor. In emergency departments (EDs) nonaccidental trauma is often falsely reported, sexual abuse is not mentioned and/or emotional abuse is not displayed or witnessed [14,15].

Systematic screening of child maltreatment in EDs did increase the recognition of suspected child maltreatment in different studies [14,16–19]. The SPUTOVAMO [14,17,18] and ESCAPE instrument [20,21] are two validated screening tools designed for the recognition of possible child maltreatment, containing the following questions about reason for visit, consistent history, injuries incompatible with history and/or developmental level of the child, inappropriate behaviour and/or interaction between child and parents, delay in seeking medical help, findings of the head-to-toe examination and other signals that make the professional doubt about the safety of the child and/or family (S1 Table and S1 Fig). When the result of the screening tool is positive, further evaluation of the child and appropriate follow-up in the context of the safety of the child should be initiated. These screening tools for children are used by ED nurses and physicians for every child who visits the ED.

Previous studies showed that children of high risk parents with risk factors such as severe psychiatric problems, substance abuse or domestic violence, are at risk of being or becoming victims of child maltreatment [22–25]. Questions designed to identify these risk factors in adult patients with children or pregnancy visiting the ED for their own condition or injury have shown to increase the identification of possible child maltreatment [26,27].

In addition to screening for signs and risk factors of child maltreatment, training and e-learning exercises for ED staff have been shown to improve the recognition of child maltreatment [19,28–30]. These specific training programmes and e-learning exercises address recognition of child maltreatment by identifying signs, how to act in case of suspicion of child maltreatment and/or communication techniques [19,28–30]. An investment of only 2 hours of e-learning for ED nurses, focused on the recognition of child maltreatment with simulations of clinical cases and video animations showed a significant improvement in the recognition of child maltreatment [28]. Also, training on communication about child maltreatment increased the application of the screening tool for child maltreatment and supported ED nurses to feel more competent [19].

For screening tools and training to be available and for the appropriate actions to be taken in case of (suspected) child maltreatment, a hospital policy regarding the recognition of child maltreatment needs to be in place [31].

Screening in combination with adequate training and hospital policy regarding child maltreatment is important to improve detection of suspected child maltreatment, subsequently take appropriate actions and protect children from further harm. To evaluate the different strategies and hospital policies for recognition of child maltreatment in EDs in Europe, and to define areas of improvement to detect child maltreatment, we conducted a survey throughout EDs in Europe. We aimed to answer the following research questions: 1) What methods for recognition of child maltreatment based on child characteristics are currently used in EDs in Europe?; 2) What methods are used for the recognition of parental risk factors related to child maltreatment in adult patients taking care of (unborn) children?; 3) What standard procedures for the recognition of child maltreatment are in place in the hospital policies of EDs in Europe?

## Methods

### Study design

All questions of the survey were developed by a multidisciplinary team, committed to effectively tackle child maltreatment and domestic violence (the European Society for Emergency Nursing (EuSEN), Research in European Paediatric Emergency Medicine (REPEM), the European Society for Emergency Medicine (EUSEM) and Augeo Foundation representatives). The survey is based on a questionnaire used by the Dutch Health Inspectorate [31] and is focused on methods used to recognize child maltreatment by addressing child and/or parental risk factors. Additionally, it addresses the presence of a hospital policy concerning child maltreatment. Several topics are questioned, including the use of screening tools, protocols and procedures, existence of training programmes, the presence of a local child maltreatment team and a child maltreatment policy officer, the registration and monitoring of suspected cases and collaboration with child protective services. The survey consists of part I, which has more general questions, and an optional part II, which has more detailed questions if the opt-out question ‘would you like to answer some more questions?’ was responded positively. Some questions of the survey allowed for multiple answers. The final version of the survey (S1 File) was tested on clarity and phrasing and adapted on the basis of feedback from experienced paediatricians and/or nurses from: CHU Tours (France), Erasmus MC (Netherlands), Haaglanden MC (Netherlands), the Augeo Foundation (Netherlands), UZ Brussel (Belgium) and the Landspitali University Hospital (Iceland).

For the distribution of the survey the following 2 methods were used:

Method 1 Key member approach:

- One key member per country of the research group of the EUSEM (adult emergency physicians) was sent an email message with the request to forward the introduction email including the survey to 5–10 emergency physicians in different general hospitals (one responsible contact person per hospital was addressed).
  The EUSEM key members represented 12 countries: the Netherlands, Italy, United Kingdom, Turkey, Sweden, Spain, Romania, France, Ireland, Denmark, Germany and Finland. Additionally, the survey was also sent to the 15 members of the French society for Emergency Medicine (SFMU) Research Committee.
- One key member per country from the REPEM network (paediatricians) was sent the same email to forward to 5–10 paediatricians/paediatric emergency physicians working in different EDs.
  The REPEM key members represented 19 countries: Austria, Belgium, France, Germany, Hungary, Israel, Italy, Latvia, Lithuania, Portugal, Qatar, Romania, Spain, Sweden, Switzerland, the Netherlands, Turkey, United Kingdom and Saudi Arabia.

Method 2 General approach:

- The EuSEN executive board asked EuSEN members to fill out the questionnaire.
  The EuSEN members are spread over at least 12 countries: Croatia, Sweden, Belgium, Norway, Greece, the Netherlands, Poland, Switzerland, Italy, Iceland, Denmark and Malta.
- At the EUSEM congress in 2018, visitors were asked through flyers, websites and presentation slides to fill out the survey.
  Researchers from all over Europe where present at the EUSEM congress.

Overall, at least 25 different countries in Europe are approached with the survey.

This study was approved by the Ethics Committee of the European Society of Emergency Medicine (EUSEM, april 16^th^ 2018). The completion of the survey by the participants was taken as consent.

### Definitions described in the survey

In the survey we described several definitions to standardize the interpretation of the survey questions:

1. A child maltreatment team is a multidisciplinary team in hospitals specializing in child maltreatment and domestic violence that meets regularly or for specific cases. The goal of the team is to ensure all employees are educated and any suspicion of child maltreatment or domestic violence is reported and addressed.
2. A child maltreatment policy officer is a hospital employee responsible for all matters related to child maltreatment.
3. The use of a screening tool can improve the recognition of child maltreatment and one of these is the ESCAPE instrument. This instrument includes the following items: consistent history, delay in seeking medical help, injury fits with developmental level, interaction, top-to-toe examination and doubt about safety.
4. Child protective services is a governmental agency responsible for providing child protection, including responding to reports of child maltreatment. Other services may include consulting a child maltreatment expert or colleague.
5. High risk parents or adult patients with parental characteristics (domestic violence, substance abuse or severe psychiatric problems) refer to situations where the parent is the patient.

### Data analysis

The distribution of the survey started in September 2018 and the last completed survey was received in January 2019. Responses from EDs in non-European countries were excluded. First, to correct for the relatively high response of Belgian EuSEN members, a random sample of the completed surveys were taken with a maximum number comparable to the highest responded country (UK).

To estimate the overall response rate, we calculated the minimum and maximum response rates in conjunction with the responses from the EUSEM and REPEM. We estimated a minimum and maximum number of potential participants from REPEM and the EUSEM of 271 and 438 responses, respectively. This calculation was based on the distribution of the survey through the different key members, each of whom contacted 5–10 professionals.

**Fig 1:**
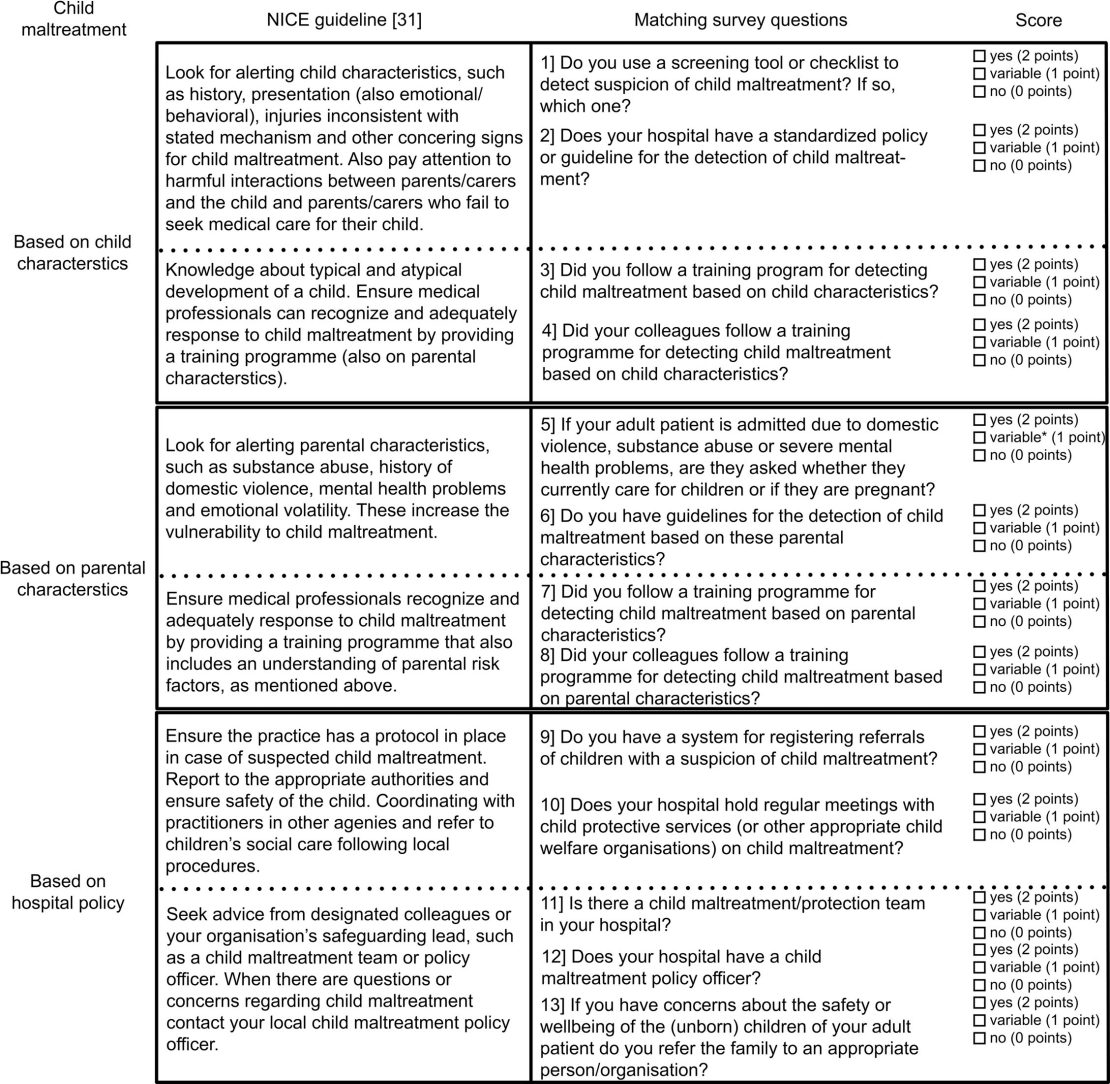
Score for recognition of child maltreatment at the ED based on the NICE guideline.

To interpret and compare the results from the survey we developed a score for the use of tools and strategies on the recognition of child maltreatment by European hospitals based on the recommendations of the NICE guideline ‘Child abuse and neglect’ [32]. The National Institute for Health and Clinical Excellence (NICE) is located in England and develops guidelines based on extensive review of the recent literature and consensus of clinicians with expertise on the subject to support international health care professionals [33]. The NICE guideline ‘Child abuse and neglect’ [32] includes recommendations for the recognition of child maltreatment, for the recognition of parental risk factors and for the presence of a hospital policy regarding child maltreatment, which were matched to our survey questions. The score included 13 items (ranging from 0–2 points per item): 4 questions concerning child characteristics, 4 questions concerning parental characteristics and 5 questions concerning hospital policy (Fig 1 and S2 Table). When respondents from the same hospital gave different answers to the same question, a ‘variable’ score (1 point) was given instead of ‘yes’ (2 points) or ‘no’ (0 points). To categorize the different hospitals into those that meet up to most, some or few NICE guideline recommendations [32] we established cut-off values of ≥75%, 50–75% and <50% of the maximum score respectively. Additionally, the score percentages were assessed per subset (child characteristics, parental characteristics and hospital policy). Responses from unknown hospitals and non-scorable surveys due to missing data were excluded. For the questions focusing on parental characteristics (questions #5, #6, #7, #8 and #13 from the score in Fig 1) responses from paediatric EDs were excluded (n = 53).

### Statistical analysis

Descriptive analyses via IBM SPSS Statistics 25 were performed. Chi-squared tests were performed to test the association of hospital type and number of ED visits with the outcome child characteristics, parental characteristics and hospital policy; and to compare the scores based on the NICE guideline of paediatric EDs and mixed/adult EDs.

## Results

**Fig 2:**
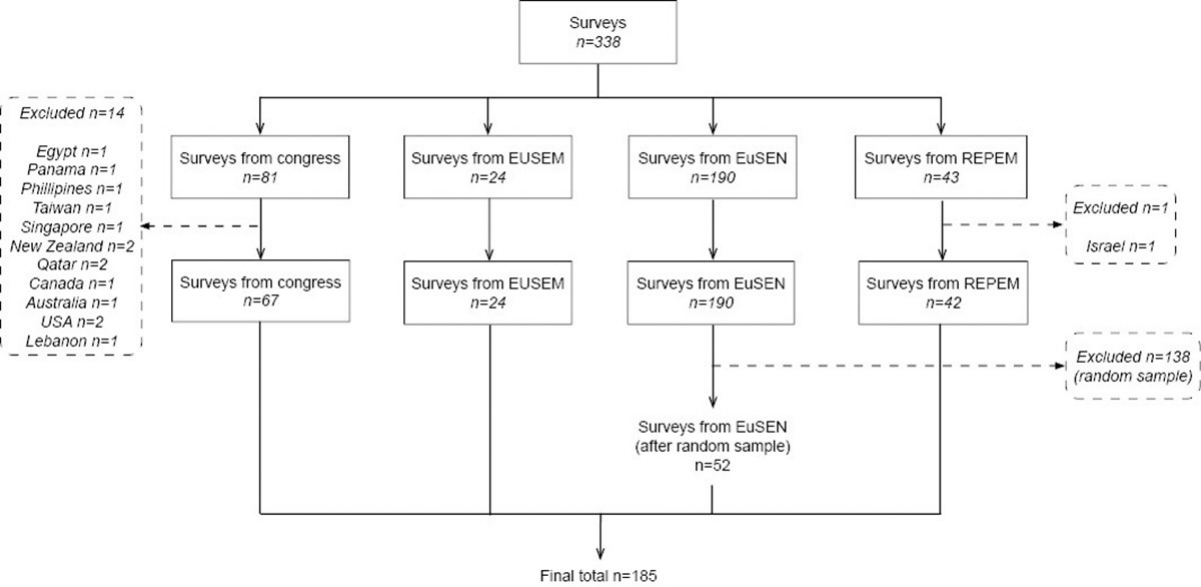
Flowchart inclusion and exclusion of the surveys. EUSEM, the European Society for Emergency Medicine; EuSEN, the European Society for Emergency Nursing; REPEM, Research in European Paediatric Emergency Medicine.

A total of 338 surveys were returned from the EUSEM congress (n = 81), the EUSEM members (n = 24), the EuSEN (n = 190) and REPEM (n = 43) (Fig 2). The estimated overall response rate from the EUSEM and REPEM taken together ranged from 33.8% (148/438) to 54.6% (148/271). Non-European responses were excluded and we excluded responses by our random sample approach for the EuSEN responses (Fig 2). The remaining 185 responses from 148 different hospitals were analysed (Table 1). Part I of the survey was completed by all respondents, while part II was completed by 67.6% of the respondents (n = 125). The responses came from 29 different countries, with more than two responses per country from 15 countries (Table 1). The characteristics of the respondents are shown in S3 Table. In total, 67.6% (125/185) of the responses came from university/teaching hospitals and 32.4% (60/185) from general hospitals. Of these hospitals 14.6% (27/185) had less than 25,000 patients, 32.4% (60/185) had 25,000 to 50,000 patients and 31.4% (58/185) had more than 50,000 patients visiting the ED in a year. Of the respondents, 20.0% (37/185) were ED nurses, 29.7% (55/185) emergency physicians, 18.9% (35/185) paediatrics emergency physicians and 14.6% (27/185) paediatricians. In total 28.6% (53/185) of the respondents worked at a paediatric ED (S3 Table).

**Table 1:** Number of respondents and different hospitals per country.

Country	Number of respondents (n)	Number of different hospitals (n)
Austria	6	5
Belgium	26	22*
Bulgaria	1	1
Croatia	2	2*
Cyprus	1	1
Czech Republic	1	1
Denmark	2	2
Estonia	1	1
France	14	14*
Germany	2	2
Greece	1	1
Hungary	3	3*
Iceland	2	2*
Ireland	7	6
Italy	9	7
Kosovo	1	1
Latvia	3	2
Lithuania	1	1
Malta	18	3*
Netherlands	10	9
Norway	1	1
Poland	1	1
Romania	2	2
Slovenia	3	3
Spain	18	11
Sweden	7	5
Switzerland	11	9
Turkey	5	5*
United Kingdom	26	25*
Total	185	148

* of which one hospital unknown.

**Fig 3:**
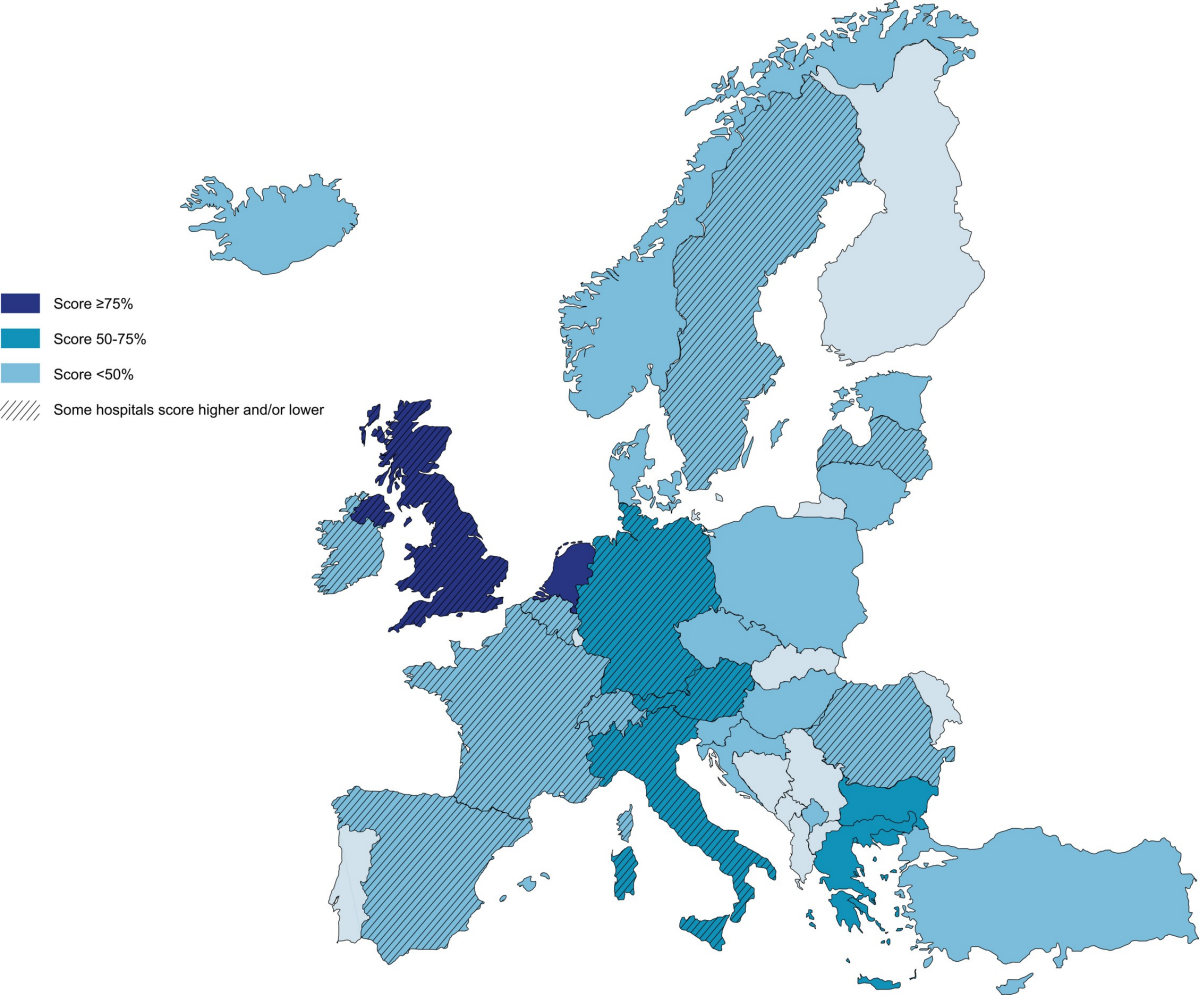
Map of recognition of child maltreatment in emergency departments in Europe. Reprinted from https://mapswire.com under a CC BY license. This image is not identical to the original image and is therefore for illustrative purposes only. See Fig 1 for subset survey questions corresponding to the presented scores.

We calculated a score based on the recommendations of the NICE guideline [32] for 133 different hospitals, of which 25.6% (34/133) had paediatric EDs (Fig 3). Scores ranged from 0% to 100% and the median total score was 46.2% (IQR 19.2%-76.9%) (Table 2).

**Table 2:** Total scores and scores of paediatric, adult and mixed EDs based on the NICE guideline.

NICE-score	Paediatric EDs n (%)	Mixed & adult EDs n (%)	Total EDs n (%)
Based on child characteristics (max. 8 points)	n = 34	n = 99	n = 133
≥75%	16 (47.1)	36 (36.4)	52 (39.1)
50–75%	5 (14.7)	15 (15.2)	20 (15.0)
<50%	13 (38.2)	48 (48.5)	61 (45.9)
Based on parental characteristics (max. 8 points)	n = 0	n = 99	n = 133
≥75%	-	28 (28.3)	28 (28.3)
50–75%	-	16 (16.2)	16 (16.2)
<50%	-	55 (55.6)	55 (55.6)
Based on hospital policy (max. 10 points^a^)	n = 34	n = 99	n = 133
≥75%	20 (58.8)	25 (25.3)	45 (33.8)
50–75%	7 (20.6)	13 (13.1)	20 (15.0)
<50%	7 (20.6)	61 (61.6)	68 (51.1)
Total score (max. 26 points^b^)	n = 34	n = 99	n = 133
≥75%	15 (44.1)	19 (19.2)	34 (25.6)
50–75%	8 (23.5)	22 (22.2)	30 (22.6)
<50%	11 (32.4)	58 (58.6)	69 (51.9)

^a^ for paediatric EDs max. points for score based on hospital policy is 8, excluding questions based on parental characteristics.

^b^ for paediatric EDs max. points for total score is 16, excluding questions based on parental characteristics.

See Fig 1 for subset survey questions corresponding to the presented scores.

### Score based on child characteristics

For the subset of questions regarding the child characteristics, 45.9% of the EDs met few (<50% of the maximum score) or none of the NICE guideline recommendations (Table 2).

The paediatric EDs scored highest on the subset of questions regarding hospital policy (p<0.001) and on the total score (0.01>p>0.001). No significant difference was found for the subset of questions regarding child characteristics (p>0.10).

In total, 28.6% of the respondents used a screening tool for child maltreatment in the ED. SPUTOVAMO, ESCAPE and local screening tools were available (Table 3). Of the hospitals using a screening tool, 48.1% (25/52) used it for all children, while 46.2% (24/52) used it only for suspected cases of child maltreatment.

**Table 3:** Detection of child maltreatment based on child characteristics, parental characteristics and hospital policy in Europe.

Child characteristics				n = 185
Screening tool or checklist				n (%)
	*Yes*			53 (28.6)
			*SPUTOVAMO*	12 (6.5)
			*ESCAPE*	5 (2.7)
			*Local screening tool*	36 (19.5)
	*No*			94 (50.8)
	*Missing*			38 (20.5)
Standardized policy or guideline				n (%)
	*Yes*			94 (50.8)
	*No*			44 (23.8)
	*Unknown*			14 (7.6)
	*Missing*			33 (17.8)
Training program for detection child maltreatment based on child characteristics^a^				n (%)
	*Yes*			81 (43.8)
			*In hospital training*	57 (30.8)
			*Regional training*	23 (12.4)
			*National training*	20 (10.8)
	*No*			78 (42.2)
	*Missing*			26 (14.1)
Training program for colleagues on detection child maltreatment based on child characteristics				n (%)
	*Yes*			67 (36.2)
	*No*			52 (28.1)
	*Unknown*			37 (20.0)
	*Missing*			29 (15.7)
Parental characteristics				n = 132
Identifying high risk parents for child maltreatment^b^				n (%)
	*Yes*			63 (47.7)
	*No*			8 (6.1)
	*Sometimes*			33 (25.0)
	*Unknown*			7 (5.3)
	*Missing/don’t see adult patients*			21 (15.9)
Guidelines for detection child maltreatment based on parental characteristics				n (%)
	*Yes*			42 (31.8)
	*No*			69 (52.3)
	*Missing/don’t see adult patients*			21 (15.9)
Training program for detection child maltreatment based on parental characteristics^a^				n (%)
	*Yes*			34 (25.8)
		*In hospital training*		28 (21.2)
		*Regional training*		6 (4.5)
		*National training*		5 (3.8)
	*No*			76 (57.6)
	*Missing*			22 (16.7)
Colleagues training program for detection child maltreatment based on parental characteristics				n (%)
	*Yes*			32 (24.2)
	*No*			50 (37.9)
	*Unknown*			27 (20.5)
	*Missing*			23 (17.4)
Hospital policy				n = 185
ED system for registering referrals of children with suspicion of child maltreatment				n (%)
	*Yes*			84 (45.4)
	*No*			43 (23.2)
	*Unknown*			16 (8.6)
	*Missing*			42 (22.7)
Regular meetings with child protective services on child maltreatment				n (%)
	*Yes*			71 (38.4)
	*No*			44 (23.8)
	*Unknown*			40 (21.6)
	*Missing*			30 (16.2)
Child maltreatment team present in hospital				n (%)
	*Yes*			82 (44.3)
	*No*			54 (29.2)
	*Unknown*			14 (7.6)
	*Missing*			35 (18.9)
Child maltreatment policy officer present in hospital				n (%)
	*Yes*			59 (31.9)
	*No*			65 (35.1)
	*Unknown*			26 (14.1)
	*Missing*			35 (18.9)
Appropriate actions taken when concerns about safety child with adult patients with parental risk factors for child maltreatment^b^ (n = 132)				n (%)
	*Yes*			92 (69.7)
	*No*			18 (13.6)
	*Missing/don’t see adult patients*			22 (16.7)

^a^ multiple answers possible.

^b^ (expecting) parents admitted due to domestic violence, substance abuse or severe mental health problems.

Half of the respondents (94/185, 50.8%), which corresponded to 54.1% (80/148) of the participating hospitals, declared they had a standardized policy or guidelines for the recognition of child maltreatment based on child characteristics. Almost half of the respondents (78/185, 42.2%) did not follow any training based on child characteristics (Table 3). Of the respondents who used a screening tool for child maltreatment, 22.6% (12/53) did not follow any training on child characteristics, and 47.2% (25/53) did not follow any training on parental characteristics. Training was mandatory for only approximately one-third of the ED nurses (59/185, 31.9%), and doctors (67/185, 36.2%) in 16 different countries, whereas in the other 13 countries training was not mandatory and/or was unknown. The need for (more) training was indicated as necessary by 133 respondents (71.9%) from 28 different countries.

Respondents from university/teaching hospitals significantly more often reported to follow training on the recognition of child maltreatment based on child characteristics (p = 0.002) than did respondents from general hospitals. Respondents from hospitals with large numbers of ED visits reported significantly more often the use of a screening tool (p = 0.001), the use of standardized guidelines (p = 0.02) and additional training on child risk factors (p = 0.003) than hospitals with less ED visits. Respondents from university/teaching hospitals did not significantly reported the need for (more) training more than respondents from general hospitals (p = 0.61), neither did respondents from hospitals with a large number of ED visits compared to less ED visits (p = 0.06).

### Score based on parental characteristics

For the subset of questions regarding parental characteristics, 55.6% (55/99) of the EDs met few or none of the NICE guideline recommendations (Table 2).

Less than one-third of the respondents (42/132, 31.8%) stated that their hospital used guidelines for detecting child maltreatment including parental risk factors. Most of the respondents did not follow any training addressing these parental characteristics (76/132, 57.6%) (Table 3). Respondents from university/teaching hospitals significantly more often reported to follow training on parental characteristics (p = 0.045) and to identify high risk parents significantly more (p = 0.002) compared to respondents from general hospitals. Respondents from hospitals with a large number of ED visits significantly more often reported to have guidelines on detection of child maltreatment based on parental characteristics (p<0.001), to follow significantly more training on parental characteristics (p<0.001) and to identify high risk parents significantly more (p = 0.006) when compared to hospitals with less ED visits.

Of all parental risk factors, domestic violence was the most common risk factor for referral to social services (92/132, 69.7%), followed by alcohol or drug overdose (80/132, 60.6%), attempted suicide (69/132, 52.3%) and other psychiatric illnesses (66/132, 50.0%) (multiple answers possible).

### Score based on hospital policy

In total, 51.1% (68/133) of the EDs met few or none of the NICE guideline recommendations on hospital policy (Table 2).

Less than half of the respondents answered that they had an ED system for registering referrals of children with suspected child maltreatment (84/185, 45.4%). Approximately one-third of the respondents declared that they had regular meetings with child protective services and had a local child maltreatment team and/or policy officer present in their hospital (Table 3).

Two-thirds of the respondents (92/132, 69.7%) stated that when high risk parents for child maltreatment are identified in the ED appropriate actions are taken for the family in case of concerns about the safety of the child(ren) and/or unborn child in case of pregnancy (Table 3).

When compared to respondents from general hospitals, respondents from university/teaching hospitals significantly more often reported: 1) to have a system for registering referrals (p = 0.003); 2) to have regular meetings with child protective services (p = 0.02); 3) to have a child maltreatment policy officer in place (p = 0.02); and 4) to take appropriate actions for children of high risk parents (p = 0.01). These differences were similar for respondents from hospitals with a large number of ED visits compared to hospitals with less ED visits (resp. p = 0.02, p = 0.003, p = 0.047 and p = 0.03). Respondents from hospitals with a large number of ED visits also significantly more often reported to have a child maltreatment team present in the hospital (p = 0.01) than hospitals with less ED visits.

Hospitals with a maximum hospital policy score ≥50% scored higher for the questions on child characteristics and parental characteristics compared to hospitals that scored <50% of the maximum on hospital policy (p<0.001 and p<0.001, respectively) (Table 4).

**Table 4:** Hospital policy score.

Score hospital policy ≥50% n (%)		Score hospital policy <50% n (%)	
Score based on child characteristics (n = 65)		Score based on child characteristics (n = 68)	
≥50%	55 (84.6)	≥50%	18 (26.5)
<50%	10 (15.4)	<50%	50 (73.5)
Score based on parental characteristics (n = 41)^a^		Score based on parental characteristics (n = 58)^a^	
≥50%	32 (78.0)	≥50%	12 (20.7)
<50%	9 (22.0)	<50%	46 (79.3)

^a^ excluding paediatric EDs (total n = 34).

## Discussion

Child maltreatment is a global problem with serious life-long consequences on health, economy and society. Improving recognition of child maltreatment followed by interventions to stop the maltreatment and provide aid and therapy will prevent children from further harm and reduce the negative impact of child maltreatment on society. Screening for signals of child maltreatment, training of hospital staff and the availability of a hospital policy regarding child maltreatment are hereby essential. Despite the availability of international guidelines, this survey shows that most hospitals (51.9%) in Europe are not sufficiently equipped to recognize child maltreatment in the ED. Paediatric EDs seem to pay substantially more attention to the recognition of child maltreatment than do mixed/adult EDs, hence complying best with the NICE guideline recommendations [32]. Similarly, university/teaching hospitals seem better equipped with respect to the recognition of child maltreatment using the strategies mentioned in comparison to general hospitals.

A quarter of the responding hospitals used a screening tool for signalling child maltreatment. Most of the responding hospitals used a local non-validated tool. It is unknown whether they are unaware of the availability of validated screening tools or whether they prefer a local non-validated tool. Louwers et al. [19] showed that the detection rate of suspected child maltreatment in children who were screened with the validated ESCAPE instrument was higher than that of children who were not screened with this tool. The performance of local tools is unknown. It might be that the use of a screening tool for every child that visits the ED reminds and empowers ED staff to critically evaluate each child visiting the ED and to consider child maltreatment even when the reason for the ED visit is or seems to be unrelated to child maltreatment [34]. Nevertheless, we must keep in mind that a screening tool only cannot replace training of hospital staff in the recognition of child maltreatment [19,30].

Training including child and parental characteristics was reported by 43.8% and 25.8%, respectively, of the respondents in contrast to more than two-thirds (71.9%) of the respondents indicating the need for (more) training. Training and/or e-learning exercises increase the confidence and knowledge of possible signs of child maltreatment and are necessary for adequately detecting and subsequently stopping child maltreatment [30]. Therefore, training should entail recognition of child maltreatment based on signs or symptoms in children and identification of risk factors in parents, such as severe psychiatric problems, substance abuse or domestic violence. Training should additionally address necessary actions to be undertaken when suspicion of child maltreatment is raised, and how to communicate with children and their parents/carers when child maltreatment is suspected [19,28–30].

The results of this survey show that there is less awareness of the recognition of parental characteristics than of child characteristics at EDs in Europe. It is known that witnessing violence, substance abuse or mental illness of any household member during childhood increases the risk for child maltreatment [22–25]. More than half of the respondents stated that they paid attention to parental risk factors in adult patients admitted to the ED, but most did not have any guidelines or any training regarding parental risk factors. Both screening for child characteristics in paediatric patients and assessing parental risk factors in adult patients caring for children are crucial for the recognition of child maltreatment.

Our results show that most European hospitals have no standard procedures or strategies, such as a child maltreatment policy officer, child maltreatment team, registering system, regular meetings with child protective services, e-learning exercises and training programmes, in place to facilitate the recognition of child maltreatment nor to monitor or refer when necessary [35–37]. In previous research, physicians stated that even though a protocol for suspected child maltreatment was present, the majority did not know where to find it or how to use it in practice [38]. In our study, some respondents working at the same hospital responded differently to questions regarding the availability of screening tools, training and policies concerning child maltreatment recognition. This indicates that often it is unclear what child maltreatment policy is present. Our results indicate that EDs with a hospital policy on child maltreatment, score better on the recognition of both child and parental characteristics in comparison to EDs that scored low on hospital policy. In general, people working in an environment with protocols and structured processes are less likely to make medical errors and do harm [39,40]. It is plausible that having a child maltreatment policy with the availability of screening tools, protocols, training and a child maltreatment team and/or officer is more likely to result in increased awareness and hence increased recognition of child maltreatment by hospital staff.

The strength of our study is that this is the first informative overview of strategies used to recognize child maltreatment by EDs in Europe in a large sample of 148 hospitals in 29 countries, and has pointed out areas of improvement.

There are also some limitations to discuss. The representativeness of the sample to reflect the actual strategies used to recognize child maltreatment at European hospitals is a known limitation to the survey design [41]. When taken into account that 67.5% of the respondents represented a teaching/university hospital, the sample might overestimate the presence of strategies to recognize child maltreatment. The responses from individual professionals are not representative of all hospital staff and different respondents within the same hospital gave varied answers, which were taken into account in the analysis.

Most of the surveys were distributed through key members (of REPEM, the EuSEN and the EUSEM), not all of whom are located in every European country. The overall estimated response rate was moderate and comparable to other surveys. Still, since non-EUSEM members could also respond via the EUSEM congress it is possible we overestimated the response rate. The number of respondents with different professions among the different countries was not always evenly distributed. We corrected the largest misdistribution (ED nurses from the EuSEN in Belgium) by a random sample, which led to a more equal representation.

In the absence of a validated score to judge quality of care, we developed an ad hoc score to assess the recognition of child maltreatment in an ED based on the recommendations of the NICE guideline [32]. We did see internal consistency in items of this score supporting its validity: when scores for hospital policy were high, child and/or parental scores were also high. However, while the NICE guideline [32] provides general information and recommendations on the recognition of child maltreatment for health professionals, no practical hands-on tools are given on how to organize this at a hospital level.

Child maltreatment is a serious public health problem and socioeconomic burden [1,2,42]. This burden includes health care costs, child welfare costs, criminal justice costs, loss of productivity, special education costs and intangible costs such as pain and suffering experienced by the affected individual and surrounding community [1,42]. In Europe, 117 million children under 18 years of age are affected by child maltreatment [1,2]. The average economic and social costs of child maltreatment in Europe were calculated to be approximately $752 billion [1,2]. In order to decrease the number of victims of child maltreatment, recognition of child maltreatment could be improved by implementation of a child maltreatment policy and should be mandatory in all hospitals in Europe. A child maltreatment policy should include a standard screening procedure and training across all hospital settings as well as the availability of a child maltreatment officer and/or team. This would support health professionals in recognizing child maltreatment, enhance adequate actions, follow-up and therapy for children and their families, and subsequently reduce further damage to these children, future generations and society. This study shows that there is much to gain in the recognition of child maltreatment in EDs in Europe. The question is not only can we do more but also should we not do more?

## Supporting information

S1 Fig Validity of signalling tools for recognition of (suspected) child maltreatment at the emergency department. (DOCX)

S1 Table Validity of signalling tools for detecting (suspected) child maltreatment at the emergency department, overview of the literature. (DOCX)

S2 Table Addendum to score for recognition of child maltreatment at the emergency department based on the NICE guideline. (DOCX)

S3 Table Characteristics of respondents on survey and of hospitals. (DOCX)

S1 File Survey on detection of family maltreatment in the emergency department. (PDF)
