# Supplementary material for: Recognition of child maltreatment in emergency departments in Europe: Should we do better?
Source: PLoS One. 2021 Feb 5;16(2):e0246361. doi: 10.1371/journal.pone.0246361 (PMC7864669; doi:10.1371/journal.pone.0246361)
Supplement: S1 Fig — (DOCX) [file pone.0246361.s001.docx]

**S1 Fig. Validity of screening tools for recognition of (suspected) child maltreatment at the emergency department**

^ PPV = positive predictive value, NPV = negative predictive value

^ Numbers 1 and 2 are the ESCAPE instrument[20,21]; 3 is SPUTOVAMO*[17]; 4 is SPUTOVAMO & TTI*[17];^10^ 5 is SPUTOVAMO-R2[14]; 6 is SPUTOVAMO-R3[14]; 7 is SPUTOVAMO-R[18].

* These estimates are an approach of the actual values, because of differential verification methods being used to verify positives and negatives
