# Supplementary material for: Recognition of child maltreatment in emergency departments in Europe: Should we do better?
Source: PLoS One. 2021 Feb 5;16(2):e0246361. doi: 10.1371/journal.pone.0246361 (PMC7864669; doi:10.1371/journal.pone.0246361)
Supplement: S1 Table — (DOCX) [file pone.0246361.s002.docx]

**S1 Table. Validity of signalling tools for detecting (suspected) child maltreatment at the emergency department, overview of the literature**

| Article | Region/  Country | Screening tool | Population | # | PPV (%) | NPV (%) | Sensitivity (%) | Specificity (%) | Comments |
| --- | --- | --- | --- | --- | --- | --- | --- | --- | --- |
| Louwers et al.; 2014[21] | Nether-lands | ESCAPE | n = 38,136 | 1 | 10  *95% CI [8.0-14.0]* | 99  *95% CI [99.7-99.9]* | 80  *95% CI [67 – 89]* | 98  *95% CI [98.0-99.0]* | Positive ESCAPE: 2.3% (n=420)  No ESCAPE: 19,861 |
| Dinpanah et al.; 2017[20] | Iran | ESCAPE | n = 6,120 | 2 | 0.34  *95% CI [0.25-0.46]* | 0  *95%CI [0-NAN]* | 100  *95% CI [87.6-100]* | 98.3  *95% CI [97.9-98.6]* | AUC = 99.2%  *95% CI [98.9-99.4]*  Positive ESCAPE: 2.2% (n=137)  Child abuse team: 35 (0.5%) |
| Teeuw et al.; 2019[17] | Nether-lands | SPUTOVAMO  TTI | n = 17,229 (admissions, 12,198 children) | 3  4 | SPUTOVAMO: 44.74  *95% CI [34.63-54.96]*  TTI: 41.10  *95% CI [29.09-54.26]*  Combination: 41.18  *95%CI [29.45-54.0]* | SPUTOVAMO: 99.70  *95% CI [99.83-99.80]*  TTI: 99.39  *95% CI [99.15-99.57]*  Combination: 99.74  *95% CI [99.52-99.86]* | *SPUTOVAMO: 78.79  *95% CI [64.84-92.74]*  *TTI: 20.00  *95% CI [-15.06-55.06]*  *Combination: 83.05  *95% CI [73.48-92.62]* | *SPUTOVAMO: 97.79  *95% CI [97.34-98.24]*  *TTI: 98.88  *95% CI [98.41-99.35]*  *Combination: 93.80  *95% CI [93.03-94.57]* | Total tested: 9,861 (57%)  Final positive diagnosis of CAN: 0.9% (107/12,198) |
| Schouten et al.; 2017[14] | Nether-lands | SPUTOVAMO-R2  SPUTOVAMO-R3 | n = 50,671 | 5  6 | 8.3  *95% CI [3.9-15.2]*  9.1  *95% BI [3.7-17.8]* | 99.1  *95% CI [98.8-99.3]*  99.1  *95% BI [98.7-99.3]* | *14.8*  *95% CI [7.0-26.2]*  *11.7*  *95% CI [4.8-22.6]* | 98.2  *95% CI [97.8-98.5]*  *98.7*  *95% CI [98.4-99.0]* | Positive checklist: 0.2% |
| Sittig et al.; 2016[18] | Nether-lands | SPUTOVAMO-R  (physical child abuse) | n = 4,290 | 7 | 3.0  *95% BI [0.6-8.5]* | 100  *95% BI [99.4-100]* | 100.0  *95% BI [29.2-100.0]* | 86.5  *95% BI [83.7-88.9]* | Prevalence physical child abuse: 0.07 *95% BI [0.01-0.2]* |
| Hoytema van Konijnen-burg et al.; 2014[34] | Nether-lands | Different local screening tools  ESCAPE  SPUTOVAMO | n = 80 hospitals | - | Not applicable | Not applicable | Not applicable | Not applicable | 16% use SPUTOVAMO,  8% use ESCAPE, 73% use local screening tools |

*These estimates are an approach of the actual values, because of differential verification methods being used to verify positives and negatives

^PPV = positive predictive value, NPV = negative predictive value
