# Supplementary material for: Recognition of child maltreatment in emergency departments in Europe: Should we do better?
Source: PLoS One. 2021 Feb 5;16(2):e0246361. doi: 10.1371/journal.pone.0246361 (PMC7864669; doi:10.1371/journal.pone.0246361)
Supplement: S2 Table — (DOCX) [file pone.0246361.s003.docx]

**S2 Table. Addendum to score for recognition of child maltreatment at the emergency department based on the NICE guideline**

| Child maltreatment | NICE guideline [32] | Derived from |
| --- | --- | --- |
| Based on the child | Look for alerting child characteristics, such as history, presentation (also emotional/behavioral), injuries inconsistent with stated mechanism and other concerning signs for child maltreatment. Also pay attention to harmful interactions between parents/carers and the child and parents/carers who fail to seek medical care for their child. | NICE guideline: see paragraphs 1.2, 1.3.12 – 1.3.44 and the paragraph ‘Alerting features for child abuse and neglect’ (p. 18). |
|  | Knowledge about typical and atypical development of a child. Ensure medical professionals can recognize and adequately response to child maltreatment, by providing a training program (also on parental characteristics). | NICE guideline: see paragraph 1.3.9 and 1.3.10. |
| Based on parental characteristics | Look for alerting parental characteristics, such as substance abuse, history of domestic violence, mental health problems and emotional volatility. These increase the vulnerability to child maltreatment. | NICE guideline: see paragraphs 1.2.3, 1.2.4 and 1.3.31 - 1.3.43 |
|  | Ensure medical professionals can recognize and adequately response to child maltreatment, by providing a training programme that also includes an understanding of parental risk factors, as mentioned above. | NICE guideline: see paragraph 1.3.10 and 1.3.11. |
| Based on hospital policy | Ensure the practice has a protocol in place in case of suspected child maltreatment. Report to the appropriate authorities and ensure safety of the child. Coordinating with practitioners in other agencies and refer to children’s social care following local procedures. | NICE guideline: see paragraphs 1.1.12, 1.3.47, 1.3.7, 1.3.10 and 1.6. |
|  | Seek advice from designated colleagues or your organisation’s safeguarding lead, such as a child maltreatment team or policy officer.  When there are questions or concerns regarding child maltreatment contact your local child maltreatment policy officer. | NICE guideline: see paragraph ‘Alerting features for child abuse and neglect’ (p.18). |
