# Supplementary material for: Recognition of child maltreatment in emergency departments in Europe: Should we do better?
Source: PLoS One. 2021 Feb 5;16(2):e0246361. doi: 10.1371/journal.pone.0246361 (PMC7864669; doi:10.1371/journal.pone.0246361)
Supplement: S3 Table — (DOCX) [file pone.0246361.s004.docx]

**S3 Table. Characteristics respondents on survey and hospitals**

| Characteristics | | Total n=185 |
| --- | --- | --- |
| Profession | | n (%) |
|  | ED Nurse | 37 (20.0) |
|  | Emergency physician | 55 (29.7) |
|  | Paediatrics emergency physician | 35 (18.9) |
|  | Resident | 13 (7.0) |
|  | Paediatrician | 27 (14.6) |
|  | ED manager | 8 (4.3) |
|  | Other | 10 (5.4) |
| Hospital type | | n (%) |
|  | General hospital | 60 (32.4) |
|  | Teaching hospital | 55 (29.7) |
|  | University/Academic hospital | 70 (37.8) |
| Hospital size | | n (%) |
|  | Small: fewer than 100 beds | 9 (4.9) |
|  | Medium: 100 to 499 beds | 76 (41.1) |
|  | Large: 500 or more beds | 100 (54.1) |
| Number of ED visits in 2017 | | n (%) |
|  | <25,000 patients | 27 (14.6) |
|  | 25,000-50,000 patients | 60 (32.4) |
|  | >50,000 patients | 58 (31.4) |
|  | Unknown | 40 (21.6) |
| Kind of patients at ED | | n (%) |
|  | Only adults | 22 (11.9) |
|  | Only children (children’s emergency department) | 53 (28.6) |
|  | Mixed: children and adults | 110 (59.5) |
