# Supplementary material for: Recognition of child maltreatment in emergency departments in Europe: Should we do better?
Source: PLoS One. 2021 Feb 5;16(2):e0246361. doi: 10.1371/journal.pone.0246361 (PMC7864669; doi:10.1371/journal.pone.0246361)
Supplement: S1 File — (PDF) [file pone.0246361.s005.pdf]

Survey: Detection of family maltreatment in the Emergency Department

Welcome to this survey

This Survey is carried out by Augeo Foundation and Erasmus MC, in collaboration with EUSEM and EUSEN. This short survey aims to gain insight into child maltreatment strategies at emergency departments (EDs) throughout Europe. Topics include: policy, use of protocols and screening tools, registration and information, training and collaboration.
The survey consists of two parts.

Part I consists of 25 general questions and takes approximately ten minutes to complete.
Part II consists of 14 more detailed questions and takes approximately five minutes to complete.

Please complete all of the questions.

Thank you in advance!
EUSEM and EuSEN boards

EUSEM, EuSEN and Augeo Foundation, Erasmus MC and Haaglanden MC from The Netherlands have joined forces to work together on improving the knowledge on child abuse and neglect and implementation of tools in order to detect more victims of family maltreatment at the ED's in Europe.

Personal information and general information about the hospital

1. Gender

- Male
- Female

2. Country

3. Profession

- ED Nurse
- Emergency physician
- Paediatric emergency physician
- Resident paediatrics
- Resident emergency medicine
- Paediatrician
- ED manager
- Other, namely

4. Years of work experience

5. Where do you work? Name and location of your organisation:

6. Hospital type:

- General
- Teaching hospital
- University / Academic

7. Funding

- Public
- Private
- Other

8. Hospital size

- Small: fewer than 100 beds
- Medium: 100 to 499 beds
- Large: 500 or more beds

Type of patients at your emergency department

With children we mean patients that are aged 17 or younger.

9. At our ED the patients are:

- ◯ Only adults
- ◯ Mixed: children and adults
- ◯ Only children (children’s emergency department)
- ◯ Unknown

Type of patients at your emergency department

10a. How many patients (adults and children) visited your hospital's ED in 2017?

- ◯ 10,000 - 25,000
- ◯ 25,000 - 50,000
- ◯ 50,000 – 100,000
- ◯ > 100,000
- ◯ Unknown

10b. Which percentage of these patients were children (0-17 years old)?

- ◯ 0% - no children
- ◯ < 10%
- ◯ 10 - 25%
- ◯ 25 - 50%
- ◯ 50 - 75%
- ◯ 100%
- ◯ Unknown

Policy, support, preconditions

11. Does your hospital have a standardized policy or guideline for the detection of child abuse?

- ◯ Yes
- ◯ No
- ◯ Unknown

Policy, support, preconditions

Child abuse/child protection team

A child abuse/child protection team is a multidisciplinary team specializing in child abuse and domestic violence that meets regularly or for specific cases. The goal of the team is to ensure that all employees are involved and that any suspicion of child abuse or domestic violence is reported and addressed.

12. Is there a **child abuse/child protection team** in your hospital?

- ◯ Yes
- ◯ No
- ◯ Unknown

Policy, support, preconditions

12a. What is the composition of the child abuse/child protection team? (Multiple answers possible)

- [ ] Paediatrician
- [ ] ED nurse
- [ ] ED doctor
- [ ] Nurse on the children’s ward
- [ ] Child psychologist
- [ ] Social worker
- [ ] Surgeon
- [ ] Unknown
- [ ] Other (please specify)

Policy, support, preconditions

Child abuse and neglect policy officer
An employee who is responsible for all matters relating to child abuse and/ or domestic violence.

13. Does your hospital have a **child abuse and neglect policy officer?**

- Yes
- No
- Unknown

13a. How often was the **child abuse and neglect policy officer** consulted by ED employees in 2017? (One answer possible)

- ◯ Never
- ◯ 1-20 times
- ◯ 21-40 times
- ◯ 41-60 times
- ◯ More than 60 times
- ◯ Unknown

Use of screening tools for children

Systematic Screening Tool for Children

Systematic screening can improve the detection rate of suspected child abuse. One of these is the ESCAPE screening tool. This instrument includes the following items: consistent history, delay in seeking medical help, injury fits with developmental level, interaction, top-toe exam, doubt about safety.

14. Do you use a **screening tool or checklist** to detect suspicion of child abuse? If so, which one?

- ◯ No standardised tool is used
- ◯ Yes, SPUTOVAMO
- ◯ Yes, ESCAPE
- ◯ Yes, local screening tool
- ◯ Other (please specify)

Use of screening tools for children

14a. When is the screening tool or checklist used?

- ◯ For all children who enter the ED
- ◯ When there is a suspicion of child abuse
- ◯ For certain risk groups (please specify)

14b. Do you know how often the screening tool or checklist was used **for children** who visited your ED in 2017?

- ◯ No
- ◯ Yes, the screening tool was used for .... % of the children

Use of child abuse protocols and screening tools for children

15. Which procedure does your hospital follow in the case of suspected child abuse?

- ☐ Contact paediatrician
- ☐ Contact child abuse and neglect policy officer
- ☐ Contact other person within the hospital
- ☐ Contact expert or organization outside the hospital
- ☐ Other (please specify)

Registration and information

Child protective services

Many countries have child protective services, a governmental agency responsible for providing child protection, including responding to reports of child abuse and neglect. Other services may include consulting a child abuse expert or colleague.

16. Does your ED have a system for registering referrals of children with a suspicion of child abuse?

- ○ Yes
- ○ No
- ○ Unknown

Use of protocols for adult patients

When treating adults with major risk factors for child abuse

Research shows that children of parents who suffer from severe psychiatric problems, abuse substances or are victims of domestic violence, have a high risk of being or becoming victims of child maltreatment.

The following questions refer to the procedure when a parent visits the ED. In other words, when the parent is the patient, not the child. The child may accompany the parent, but in most cases the ED professional will not have seen the child.

17. If your adult patient is admitted due to domestic violence, substance abuse or severe mental health problems, are they asked whether they currently care for children or if they are pregnant?

- ○ Yes
- ○ No
- ○ Sometimes

18. If you have concerns about the safety or well-being of the (unborn) children of your adult patient do you refer the family to an appropriate person/organisation?

- ○ Yes
- ○ No

19. Do you have guidelines for the detection of child maltreatment based on these parental characteristics?

- ○ Yes
- ○ No

Use of protocols when adults are your patients

19a. Which parental risk factor would prompt you to you refer children to social services? (Multiple answers possible)

- ☐ Domestic violence
- ☐ Attempted suicide
- ☐ Alcohol/drug overdose
- ☐ Other (please specify)

Registration and information

20. Does your ED register referrals of families to the Advice and Reporting Centre for Child Abuse based on parental characteristics?

- ◯ No
- ◯ Unknown
- ◯ Yes, in the patient's medical record
- ◯ Yes, in a separate system
- ◯ Yes, (please specify)

Training to detect child abuse

Training programme

Professionals who have been trained in how to detect child abuse are more likely to take action if they have concerns about a particular family.

21a. Did you follow a training programme for detecting child abuse based on **child characteristics?**

- [ ] Yes, in-hospital training
- [ ] Yes, regional training
- [ ] Yes, national training
- [ ] No

21b. Did you follow a training programme for detecting child abuse based on **parental characteristics?** (Multiple answers possible)

- [ ] Yes, in-hospital training
- [ ] Yes, regional training
- [ ] Yes, national training
- [ ] No

Training in detecting child abuse

22a. Did your colleagues follow a training programme for detecting child abuse based on child characteristics?

- ◯ Yes
- ◯ No
- ◯ Unknown

22b. Did your colleagues follow a training programme for detecting child abuse based on parental characteristics?

- ◯ Yes
- ◯ No
- ◯ Unknown

22c. Which employees are offered a training programme for detecting child abuse? (Multiple answers possible)

- ☐ ED Nurses
- ☐ Paediatric residents
- ☐ Emergency medicine residents
- ☐ Other resident
- ☐ Emergency physicians
- ☐ Paediatricians
- ☐ Surgeons
- ☐ Unknown
- ☐ Other, please specify

Training in detecting child abuse

In the following questions, 'trained to detect child abuse' refers to detection based on injuries, behaviour and characteristics of children and on the situation, behaviour and condition of parents (without seeing the children).

23a. Is it mandatory for **ED nurses** to be trained to detect child abuse?

- ◯ Yes
- ◯ No
- ◯ Unknown

23b. Is it mandatory for **ED doctors** to be trained to detect child abuse?

- ◯ Yes
- ◯ No
- ◯ Unknown

24. Is there a need for (more) training?

- ◯ Yes
- ◯ No
- ◯ Unknown

Collaboration

25. Does your hospital hold regular meetings with child protective services (or other appropriate child welfare organisations) on child abuse?

- ◯ Yes
- ◯ No
- ◯ Unknown

Other remarks

End op Part I

You have now reached the end of Part I of the survey 'Detection of family maltreatment in the emergency department'. Thank you for answering these questions.
We would appreciate it if you could complete Part II as well.

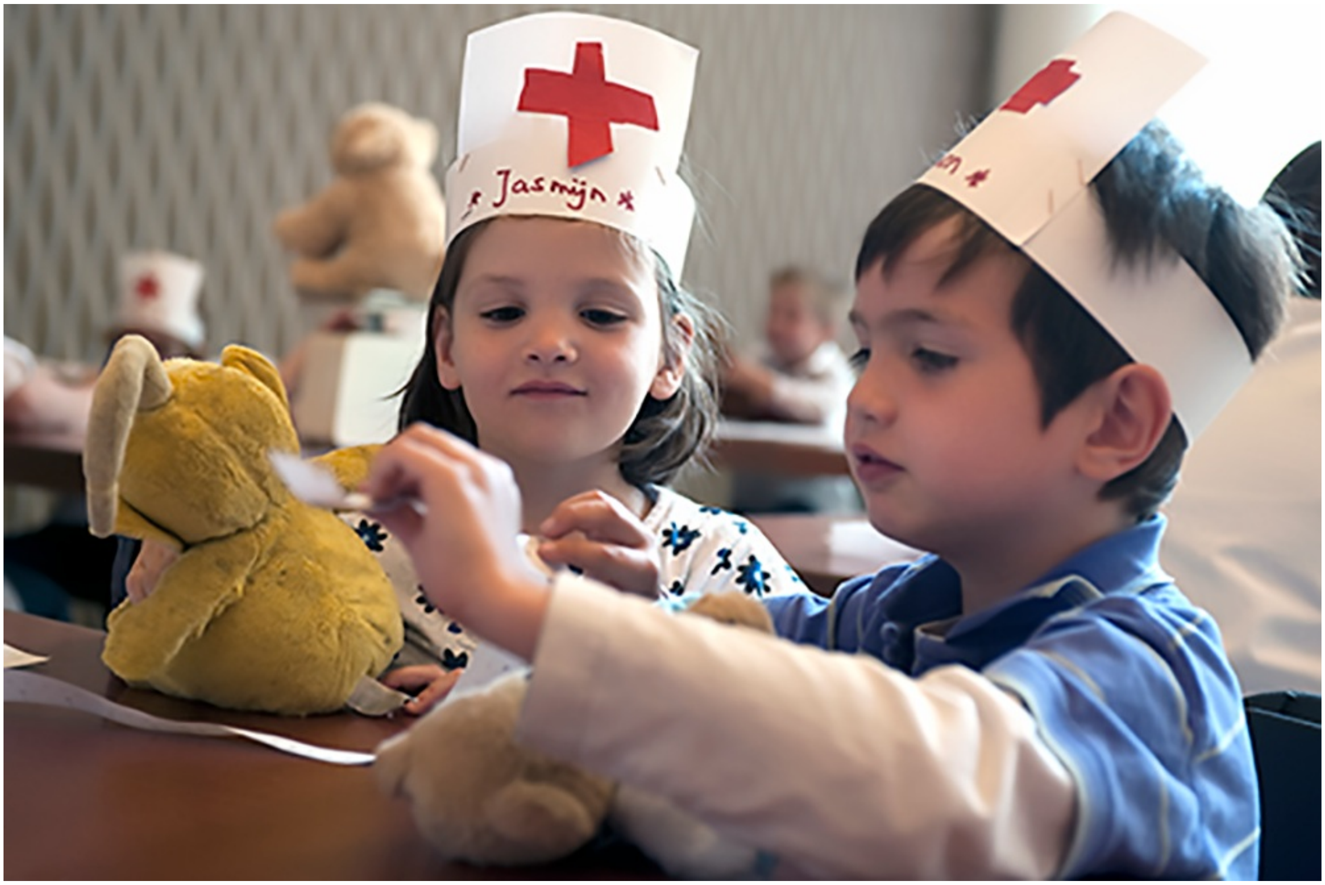

Part II: Detection of family maltreatment in the emergency department.

Would you like to answer some more questions?

- ◯ Yes
- ◯ No

Part II: Policy, support, preconditions

26. Has the policy or guideline for dealing with children in the ED, been established in writing?

- Yes
- No
- Unknown
- We have no policy or guideline

27. Have the objectives, tasks and/or procedures of the child abuse/child protection team been established in writing?

- Yes
- No
- Unknown
- We have no child abuse/child protection team

28. What is the profession of the child abuse and neglect policy officer in the hospital? (Multiple answers possible)

- Paediatrician
- Social worker
- Child psychologist
- Nurse
- We don't have a child abuse special-task officer
- Other (please specify)

29. How often does the **child abuse/child protection team** meet?

- ☐ Regularly
- ☐ At the moment when there is a specific case
- ☐ Never
- ☐ Unknown
- ☐ We have no child abuse/child protection team
- ☐ Other, namely

29a. How often does the **child abuse/ child protection team** meet?

- ◯ At least once a week
- ◯ At least twice a month
- ◯ At least once a month
- ◯ At least once a quarter
- ◯ At least once a year

Part II: Protocols for adult patients

Parents as patients

Research shows that children of parents who suffer from severe psychiatric problems, substance abuse or are victims of domestic violence have a high risk of being or becoming victims of child maltreatment. The following questions refer to the procedure when a parent visits the ED. In other words, when the parent is the patient not the child. The child may accompany the parent, but in most cases the ED professional will not see the child.

30. To whom are cases of domestic violence referred?

- ◯ To an external organisation
- ◯ The hospital's child abuse/child protection team
- ◯ No standard referral
- ◯ Unknown

31. If you refer directly to an external organisation, have clear arrangements been made with this organization on how the ED refers parents?

- ◯ Yes
- ◯ No
- ◯ Unknown

32. Do you receive feedback from the referral organization or individual concerning the follow-up after a parental referral?

- Yes
- No
- Sometimes
- Other (please specify)

33. If feedback is sent, is it added to the patient's medical record?

- Yes
- No
- Sometimes
- Other (please specify)

Part II: Training in detecting child abuse

34. How often is training on child abuse offered to ED nurses and doctors?

- ◯ Part of the training curriculum of the ED
- ◯ Yearly
- ◯ Occasionally
- ◯ We don't have a training programme for recognizing and reporting child abuse
- ◯ Other frequency (please specify)

35. If training is offered, how long does it last?

- ◯ < 1 hour
- ◯ 1-2 hours
- ◯ More than 2 hours

36a. Could you estimate the percentage of current **ED nurses** who have followed a training programme for detecting child abuse over the last 3 years?

- ◯ Most
- ◯ Half
- ◯ Small part
- ◯ None

36b. Could you estimate the percentage of current ED residents who have followed a training programme for detecting child abuse over the last 3 years?

- ◯ Most
- ◯ Half
- ◯ Small part
- ◯ None

Part II: Collaboration

37. How often are meetings on child abuse held between the hospital and child protective services?

- ◯ Never
- ◯ Less than once a year
- ◯ At least once a year
- ◯ At least once every 6 months
- ◯ At least once every 2 months

38. If structural meetings are held, who attends these meetings with child protective services? (Multiple answers possible)

- ☐ Paediatrician
- ☐ ED nurse
- ☐ Head of ED
- ☐ Children's nursing ward
- ☐ Child psychologist
- ☐ Social worker
- ☐ Surgeon
- ☐ Child abuse/child protection team
- ☐ We have no structural meetings

You have reached the end of the survey

Do you have any questions or comments with regard to this survey?

We would like to contact you to inform you of the results and to ask any additional follow-up questions. Please leave your name and contact details (e-mail address). *

Name:

E-mail address:

* We will only use your personal data for the purposes described above and in compliance with the General Data Protection Regulation
